# Factors associated with regional retention of physicians: a cross-sectional online survey of medical students and graduates in Japan

**DOI:** 10.1186/s12960-023-00871-z

**Published:** 2023-10-26

**Authors:** Soichi Koike, Kentaro Okazaki, Akiko Tokinobu, Masatoshi Matsumoto, Kazuhiko Kotani, Hitomi Kataoka

**Affiliations:** 1 Division of Health Policy and Management, Center for Community Medicine, Jichi Medical University, 3311-1 Yakushiji, Shimotsuke, Tochigi 329-0498 Japan; 2 Department of Education for Community-Oriented Medicine, Nagoya University Graduate School of Medicine, 65 Tsurumai-Cho, Showa-Ku, Nagoya, Aichi 466-8550 Japan; 3 Faculty of Medical Sciences Community Medicine Education Unit, Kyushu University Graduate School of Medical Sciences, 3-1-1 Maidashi, Higashi-Ku, Fukuoka, 812-8582 Japan; 4 Center for Diversity and Inclusion, Okayama University Hospital, Okayama University, 2-5-1 Shikata-Cho, Kita-Ku, Okayama 700-8558 Japan; 5 Department of Community-Based Medical System, Graduate School of Biomedical and Health Sciences, Hiroshima University, 1-2-3 Kasumi, Minami-Ku, Hiroshima 734-8553 Japan; 6 Division of Community and Family Medicine, Center for Community Medicine, Jichi Medical University, 3311-1 Yakushiji, Shimotsuke, Tochigi 329-0498 Japan; 7 Center for Medical Education and Internationalization, Kyoto University, Yoshida-Konoe-Cho, Sakyo-Ku, Kyoto 606-0007 Japan

**Keywords:** Regional retention, Physician maldistribution, Regional quota, Medical education, Career development program

## Abstract

**Background:**

Physician shortage and maldistribution is an urgent health policy issue requiring resolution. Determination of factors associated with regional retention and development of effective policy interventions will help to solve this issue. The purpose of the present study was to identify factors associated with regional retention and discuss their policy implications.

**Methods:**

We conducted a cross-sectional online survey from February to March of 2022 for graduates from regional quotas (special quotas for medical schools to select students engaged in community medicine) and Jichi Medical University (JMU) and students at 10 medical schools including JMU. Completed surveys were obtained from 375 graduates and 1153 students. Questions included intention to continue to work in their home prefecture in the future, as well as background information and potential factors associated with regional retention. In the analyses, regional quotas and JMU were referred to as community medicine-oriented programs and schools (CMPS). We performed logistic regression analyses to identify factors associated with regional retention.

**Results:**

Among the students, scholarship-bonded obligatory service, satisfaction with current life, intention to belong to *ikyoku* (a traditional physician allocation/training system in Japanese medical schools), and interest in general practice/family medicine were significantly positively associated with regional retention. Among the graduates, satisfaction with training environment, intention to belong to *ikyoku*, and recommending their program to high school students were significantly positively associated with regional retention. For students of CMPS, satisfaction with the career development program was positively associated with future regional retention. For graduates, this association was observed only in the crude analysis.

**Conclusions:**

In addition to known factors such as interest in general practice/family medicine, intention to belong to *ikyoku* had a substantial impact on regional retention. The present results suggest that the career support system represented by *ikyoku* as well as a career development program are of potential importance for increasing regional retention through the mechanisms of a sense of belonging and a life-long education system. These findings provide useful information for the development of further policy interventions that interweave traditional and new systems to maximize their effectiveness.

## Background

Physician shortage and maldistribution is one of the urgent health policy issues requiring resolution [1]. Determination of factors associated with regional retention of physicians and development of effective policy interventions will assist in solving this issue.

Many studies have been conducted to identify factors that contribute to the recruitment and retention of physicians in medically underserved regions and communities. Original attributes (nature) and educational perspectives (nurture) are used as frameworks for these studies [2]. Regarding the nature of physicians, originating from a rural area was strongly associated with a desire to work in a rural area, and actual work location as well as being interested in a comprehensive specialty were related to primary care [3]. For the nurture of education, medical students who have experienced a long period of rural training and physicians who have had both middle and high school education and training in the same rural area were likely to remain in the same rural area after training [4]. The salmon homecoming theory, which states that people educated in rural areas often work in rural areas, is also well known [5].

Various policies to secure physicians are in place in different countries. For example, Thomas Jefferson University initiated the Physician Shortage Area Program in 1974. The program selectively admits medical school students who both grew up in and plan to practice in a rural area. The program contributed 12% of all rural family physicians in Pennsylvania and helped to achieve > 70% long-term physician retention in rural family medicine after 20–25 years [6]. In Thailand, the government has implemented a multi-pronged intervention strategy over several decades to attract and retain doctors in underserved areas, including a special track for recruitment and training that enrolls students with rural backgrounds, trains the students at medical schools and hospitals close to their home towns, and obliges the students to return to their home provinces upon graduation. This track currently accounts for 47% of the total number of new graduates for general practice [7].

The World Health Organization published policy guidelines and recommendations in 2010 [1]. Among the suggested measures, one of the most frequently used approaches is a compulsory placement program, which is implemented in 70 countries [8]. However, there is a limited reliable evidence for the effects of interventions to address the inequitable distribution of health professionals [9], and the evidence is mixed for financial incentives and return of service programs [10, 11].

The issue of uneven distribution and availability of physicians is also a major health policy issue in Japan. Past empirical research has shown that simply increasing the number of physicians is not sufficient to mitigate the maldistribution of physicians [12, 13]. Consequently, there are two major approaches to increase the number of physicians working in the community. One is to establish a medical school that produces physicians for rural medicine (Jichi Medical University [JMU]), and the other is to allocate certain entrance quotas for medical schools to select students engaged in community medicine (regional quotas).

JMU was founded in 1972. Its budget is derived from the national government, as well as all 47 prefectural governments. Several entrance quotas are set for each prefecture. The JMU undergraduate education program is designed to focus on community and rural medicine, as well as other areas of medicine. After students have passed their national medical license and completed a 9-year obligation period including several years of rural service, the tuition fees are waived [3]. A previous study confirmed that JMU graduates who completed their obligation period were four times more likely to work in rural areas than non-JMU graduates [14].

Regarding regional quotas, although the programs vary, most contain at least one of the following components: applicants should have a geographical background in the prefecture where the medical school is located; applicants should undertake a special admission process with an emphasis on their motivation to commit to community medicine in their prefecture; applicants should have more exposure to community-based practice in their undergraduate medical education; and upon graduation, applicants are obliged or expected to work in the prefecture for several years [15]. Most of the regional quota programs are bundled with a scholarship, and in exchange, the graduates must work in the prefecture for a certain period of time. In most programs, one-third to one-half of the required period is dedicated to working in a rural area within the prefecture. Many programs offer special undergraduate curricula and programs. The percentage of medical school enrollment for regional quotas has increased rapidly, reaching 1,723 places, or 18.7% of the enrollment capacity of all medical schools in fiscal year 2021 [16].

In addition to being community medicine-oriented, one of the common features of JMU and regional quotas is the introduction and application of a career development program developed by each prefecture. From the physician’s point of view, the obligation to work in a rural area for several years after graduation coincides with a critical period in their career development pathway, and thus it is an important issue how to balance their scholarship-bonded rural service obligation, career development, and other major life events, such as marriage and child-raising, that are often experienced in the same life stage. The introduction of a career development program is designed to solve this dilemma by providing multiple courses for each area of practice and type of medical institution where the physicians work and by visualizing the career paths that can be undertaken in each course including the board certification that can be obtained.

As such, the regional quotas and JMU have much in common and play major roles in securing physicians in community medicine and rural regions. However, there are also differences between the two approaches. The retention rate for contractual rural service was higher among JMU graduates than among regional quota graduates with a scholarship [17]. It was also shown that a higher percentage of physicians from regional quotas work in non-urban areas compared with physicians in general [15]. It was documented that students within regional quotas become less willing to work in the region as the academic year progresses [18]. Meanwhile, the cost for prefecture for JMU was higher than that for regional quotas [17]. Thus, how to combine these two approaches and determine ways to retain medical school graduates in community medicine and rural regions remains an important issue.

Historically, the Japanese medical specialist system has been operated independently by individual academic societies, and there have been concerns about accreditation standards and quality assurance. In 2013, a national panel recommended the establishment of a third-party organization to unify the evaluation and accreditation of medical specialists and training programs. A new board certification system established general practice as one of the 19 basic specialties. In Japan, general practice and family medicine remain unpopular, and specialists also provide primary care [19]. In this regard, the change in policy has the potential to alter the mode of medical provision. A new training system for board certification was launched in 2018. Nevertheless, the number of students who commenced training to become a board-certified general practitioner in 2023 was only 285, or 3.1% of the 9,325 students who began training in any one of the basic specialties [20].

To mitigate physician maldistribution, it is also important to consider the placement mechanism of physicians. In this regard, *ikyoku*, a historical and traditional system for physician allocation, should be taken into account. During the modernization process in Japan, the training and personnel system for doctors based on *ikyoku* (literal translation: the clinical department of a medical school characterized by a professor at the top of the hierarchy) was imported from Germany. Combined with the traditional Japanese apprentice system and the spirit of craftsmanship, the system in Japan has developed in its own way. Its unique feature is the power of professors in university hospitals to rotate physicians among affiliated hospitals [19, 21]. The Japanese postgraduate medical education system is regarded as an apprenticeship-based system [22], with most new graduates trained in a medical school and belonging to that school. Even after their residency is completed, the relationship continues [23]. The physicians in most larger hospitals remain under the influence of this system.

Meanwhile, little is known about the actual conditions and contributing factors that influence the intention to work in rural regions and community medicine, especially with a focus on career development. Therefore, the purpose of the present study was to identify factors associated with regional retention and to discuss their policy implications.

## Methods

We conducted a cross-sectional online questionnaire survey from February to March 2022. In the study, JMU and regional quotas were referred to as community medicine-oriented programs and schools (CMPS), taking into account their common features of community medicine-oriented approach and application of career development programs.

**Fig. 1:**
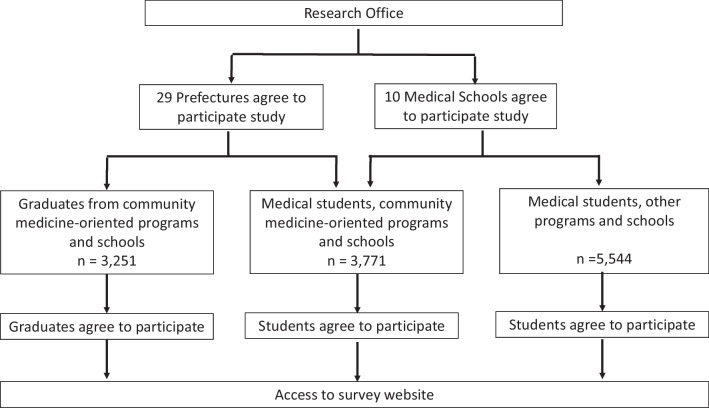
Overview of the study design

The study subjects were medical students and graduates of CMPS and medical students in other programs and schools. Requests for cooperation with the study were sent to graduates of JMU and regional quotas within the scholarship-bonded obligatory rural service period through their prefectures. Among the 47 prefectures in Japan, 29 prefectures agreed to forward our recruiting email. Ten medical schools (9 medical universities with regional quotas [Akita University, Niigata University, Nagoya University, Okayama University, Hiroshima University, Kochi University, Nagasaki University, Saga University and Kagoshima University] and JMU) forwarded the recruiting email to their medical students. Subjects who received the recruiting email and agreed to participate in the survey accessed the online survey site. The survey was conducted anonymously (Fig. 1).

**Fig. 2:**
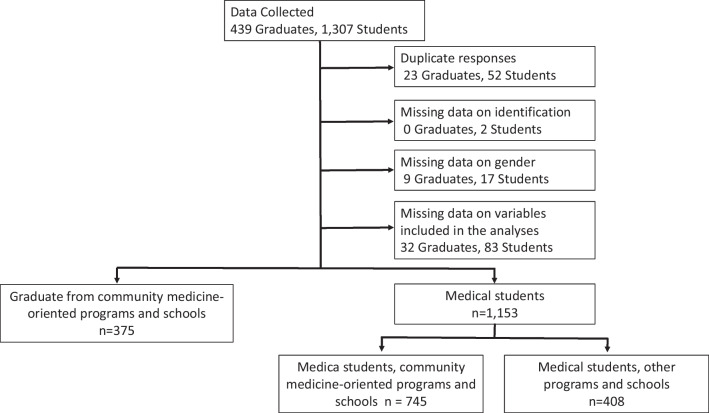
Selection of the study participants

By the closing date of the online survey, 1,746 respondents (physicians: *n* = 439; students: *n* = 1307) were identified among the 3251 physicians, and 9315 students expected to receive the recruiting email. Among the respondents, 218 were excluded because of duplicate responses or missing data on student/physician identification, sex, or variables included in the analyses, leaving 1,528 respondents (physicians: *n* = 375; students: *n* = 1153) for the analyses (Fig. 2).

The question items for all participants included their prefecture of origin, sex, current academic year or years since graduation, marital status, family structure (presence or absence of children), whether or not their family members were medical practitioners, future prospect for area of practice, intention for regional retention, thoughts for career development, and ideas on regional medicine. For the students and graduates from CMPS, further questions were asked about the prefecture for completion of their obligation period. For graduates, type of workplace, place of practice, training environment, regional assignment, starting year of regional service after graduation, and expected duration of regional service were also asked. We developed the online questionnaire with the above question items using the online survey tool SurveyMonkey (Momentive Inc., San Mateo, CA, USA).

We performed logistic regression analyses and estimated the crude and controlled odds ratios (ORs) and their 95% confidence intervals (CIs) to identify factors associated with regional retention.

The dependent variable was regional retention, measured with the related survey question “I intend to work in my current prefecture for a long time in the future.” The question was answered on a 5-point Likert scale, ranging from 5 for strongly agree to 1 for strongly disagree. We categorized responses 4 and 5 as “yes” and responses 1, 2, 3, and “not sure” as “no” for the analyses.

Independent variables were selected as those considered to be potential factors associated with regional retention. We dichotomized the responses to Likert-scale questions as follows: for 5-point Likert scale questions, responses 4 and 5 were categorized as “yes” and other responses as “no”; for 10-point Likert scale questions, responses above the median were categorized as “yes” and other responses as “no”. Among the variables, satisfaction with current student life/current life as physician/present training environment, intention of belong to *ikyoku* (a traditional physician allocation/training system in Japanese medical schools), interest in general practice/family medicine, recommending their program to high school students, and satisfaction with education or activities specially designed for their program were questions answered on a 5-point Likert scale. Appreciation of career development program was answered on a 10-point Likert scale. In the study, median and above meant ≥ 8 for students and ≥ 7 for graduates.

In the statistical analyses, two-sided *p*-values less than 0.05 were considered statistically significant. All analyses were conducted using Stata SE 17.0 for Windows (Stata Corp., College Station, TX, USA).

## Results

Table 1 shows the characteristics of the study participants. Students of CMPS showed lower proportions of male students, and children of medical practitioners, and a higher proportion chose pediatrics and general practice as their future prospect than students in other programs and schools. A lower proportion of graduates from CMPS chose general practice as their future prospect compared with medical students.

**Table 1:** Characteristics of the study participants

	Medical students			CMPS graduates	Total
	CMPS	Others	Subtotal		
Number of study participants	745	408	1153	375	1528
Sex					
Male, *n* (%)	365 (49.0)	246 (60.3)	611 (53.0)	238 (63.5)	849 (55.6)
Medical student grade/PGY, *n* (%)					
1st year/PGY 1	163 (21.9)	75 (18.4)	238 (20.6)	52 (13.9)	
2nd year/PGY 2	142 (19.1)	77 (18.9)	219 (19.0)	54 (14.4)	
3rd year/PGY 3	129 (17.3)	66 (16.2)	195 (16.9)	49 (13.1)	
4th year/PGY 4	102 (13.7)	80 (19.6)	182 (15.8)	55 (14.7)	
5th year/PGY 5	130 (17.4)	65 (15.9)	195 (16.9)	44 (11.7)	
6th year/PGY 6	79 (10.6)	45 (11.0)	124 (10.8)	38 (10.1)	
PGY 7				40 (10.7)	
PGY 8				29 (7.7)	
PGY 9				14 (3.7)	
Marital status, *n* (%)					
Married	10 (1.3)	11 (2.7)	21 (1.8)	199 (53.1)	220 (14.4)
Not married	725 (97.3)	389 (95.3)	1114 (96.6)	169 (45.1)	1283 (84.0)
Others, no response	10 (1.3)	8 (2.0)	18 (1.6)	7 (1.9)	25 (1.6)
Number of children, *n* (%)					
One or more	0 (0.0)	4 (1.0)	4 (0.4)	118 (31.5)	122 (8.0)
None	742 (99.6)	403 (98.8)	1145 (99.3)	256 (68.3)	1401 (91.7)
No response	3 (0.4)	1 (0.3)	4 (0.4)	1 (0.3)	5 (0.3)
Children of medical practitioners, *n* (%)					
Yes	57 (7.7)	47 (11.5)	104 (9.0)	30 (8.0)	134 (8.8)
No	679 (91.1)	355 (87.0)	1034 (89.7)	342 (91.2)	1376 (90.1)
No response	9 (1.2)	6 (1.5)	15 (1.3)	3 (0.8)	18 (1.2)
Future prospects (multiple response), *n* (%)					
Internal medicine	385 (51.7)	192 (47.1)	577 (50.0)	135 (36.0)	712 (46.6)
Pediatrics	237 (31.8)	95 (23.3)	332 (28.8)	43 (11.5)	375 (24.5)
Dermatology	43 (5.8)	38 (9.3)	81 (7.0)	9 (2.4)	90 (5.9)
Psychiatry	62 (8.3)	44 (10.8)	106 (9.2)	10 (2.7)	116 (7.6)
Surgery	133 (17.9)	98 (24.0)	231 (20.0)	35 (9.3)	266 (17.4)
Orthopedics	94 (12.6)	59 (14.5)	153 (13.3)	23 (6.1)	176 (11.5)
Obstetrics and gynecology	146 (19.6)	63 (15.4)	209 (18.1)	36 (9.6)	245 (16.0)
Ophthalmology	33 (4.4)	23 (5.7)	56 (4.9)	6 (1.6)	62 (4.1)
Otolaryngology	30 (4.0)	26 (6.4)	56 (4.9)	5 (1.3)	61 (4.0)
Urology	27 (3.6)	17 (4.2)	44 (3.8)	10 (2.7)	54 (3.5)
Neurosurgery	45 (6.0)	32 (7.8)	77 (6.7)	10 (2.7)	87 (6.0)
Radiology	24 (3.2)	28 (6.9)	52 (4.5)	4 (1.1)	56 (3.7)
Anesthesiology	73 (9.8)	56 (13.7)	129 (11.2)	12 (3.2)	141 (9.2)
Pathology	20 (2.7)	22 (5.4)	42 (3.6)	7 (1.9)	49 (3.2)
Laboratory medicine	6 (0.8)	7 (1.7)	13 (1.1)	0 (0.0)	13 (0.9)
Emergency medicine	149 (20.0)	75 (18.4)	224 (19.4)	34 (9.1)	258 (16.9)
Plastic surgery	17 (2.3)	29 (7.1)	46 (4.0)	2 (0.5)	48 (3.1)
Rehabilitation	17 (2.3)	16 (3.9)	33 (2.9)	11 (2.9)	44 (2.9)
General practice	295 (39.6)	79 (19.4)	374 (32.4)	34 (9.1)	408 (26.7)
Others, no response	29 (3.9)	32 (7.8)	61 (5.3)	18 (4.8)	79 (5.2)
Type of medical facility, *n* (%)					
University hospital	–	–	–	24 (6.4)	–
Non-university hospital (≥ 200 beds)	–	–	–	50 (13.3)	–
Non-university hospital (< 200 beds)	–	–	–	173 (46.1)	–
Clinic	–	–	–	124 (33.1)	–
Others	–	–	–	4 (1.1)	–
Practice location, *n* (%)					
Large city or its suburbs	–	–	–	59 (15.7)	–
Regional city or its suburbs	–	–	–	242 (64.5)	–
Remote area or island	–	–	–	73 (19.5)	–
Others	–	–	–	1 (0.3)	–

*CMPS* community medicine-oriented programs and schools, *PGY* post graduate year

Table 2 shows the crude and controlled ORs and their 95% CIs for regional retention in relation to the selected factors among the medical students. The factors of scholarship-bonded obligatory rural service, satisfaction with current student life, intention to belong to *ikyoku*, and interest in general practice/family medicine were significantly positively associated with regional retention.

**Table 2:** Factors for regional retention among the medical students from all categories (*n* = 1153)

Variables	Prevalence *n* (%)	Crude OR (95% CI)	Controlled OR (95% CI)
Scholarship-bonded obligatory rural service			
No	108/408 (26.5)	Reference	Reference
Yes	557/745 (74.8)	8.23 (6.25–10.84)	8.33 (6.17–11.23)
Sex			
Male	344/611 (56.3)	Reference	Reference
Female	321/542 (59.2)	1.13 (0.89–1.43)	0.86 (0.65–1.14)
Satisfaction with current student life			
Not satisfied	239/502 (47.6)	Reference	Reference
Satisfied	426/651 (65.4)	2.08 (1.64–2.64)	1.77 (1.34–2.34)
Intention of belonging to *ikyoku*			
No	373/740 (50.4)	Reference	Reference
Yes	292/413 (70.7)	2.37 (1.84–3.07)	3.00 (2.21–4.08)
Interest in general practice/family medicine			
No	279/599 (46.6)	Reference	Reference
Yes	386/554 (69.7)	2.64 (2.07–3.36)	2.14 (1.61–2.83)

*OR* odds ratio, *CI* confidence interval

Table 3 shows the crude and controlled ORs and their 95% CIs for regional retention in relation to selected factors among the medical students with scholarship-bonded obligatory rural service. The controlled ORs of the factors revealed positive associations with regional retention for satisfaction with current student life, intention to belong to *ikyoku*, interest in general practice/family medicine, recommending their program to high school students, and appreciation of the career development program. An association between regional retention and satisfaction with education or activities especially designed for the respondent’s program was observed in the crude analysis.

**Table 3:** Factors for regional retention among the medical students of community-medicine oriented programs and school (*n* = 745)

Variables	Prevalence *n* (%)	Crude OR (95% CI)	Controlled OR (95% CI)
Sex			
Male	276/365 (75.6)	Reference	Reference
Female	281/380 (74.0)	0.92 (0.66–1.27)	0.95 (0.67–1.36)
Satisfaction with current student life			
Not satisfied	192/296 (64.9)	Reference	Reference
Satisfied	365/449 (81.3)	2.35 (1.68–3.30)	1.81 (1.25–2.62)
Intention of belonging to *ikyoku*			
No	324/474 (68.4)	Reference	Reference
Yes	233/271 (86.0)	2.84 (1.91–4.21)	2.99 (1.98–4.51)
Interest in general practice/family medicine			
No	223/329 (67.8)	Reference	Reference
Yes	334/416 (80.3)	1.94 (1.39–2.70)	1.76 (1.22–2.54)
Recommending your program to high school students			
No	279/416 (67.1)	Reference	Reference
Yes	278/329 (84.5)	2.68 (1.86–3.84)	2.09 (1.39–3.14)
Satisfaction with education or activities specially designed for your program at medical school			
Not satisfied	284/407 (69.8)	Reference	Reference
Satisfied	273/338 (80.8)	1.82 (1.29–2.56)	0.95 (0.63–1.42)
Appreciation of the career development program			
No	351/502 (69.9)	Reference	Reference
Yes	206/243 (84.8)	2.40 (1.61–3.57)	1.66 (1.08–2.56)

*OR* odds ratio, *CI* confidence interval

The factors that were positively associated with regional retention among the CMPS graduates were satisfaction with present training environment, intention to belong to *ikyoku*, and recommending their program to high school students (Table 4). The factors of satisfaction with current life as a physician and appreciation of the career development program were not significantly associated with regional retention when controlled for other factors.

**Table 4:** Factors for regional retention among the graduates of community-medicine oriented programs and school (*n* = 375)

Variables	Prevalence *n* (%)	Crude OR (95% CI)	Controlled OR (95% CI)
Sex			
Male	172/238 (72.3)	Reference	Reference
Female	94/137 (68.6)	0.84 (0.53–1.33)	0.86 (0.52–1.42)
Satisfaction with present training environment			
Not satisfied	79/144 (54.9)	Reference	Reference
Satisfied	187/231 (81.0)	3.50 (2.20–5.56)	2.37 (1.36–4.11)
Satisfaction with current life as a physician			
No	85/144 (59.0)	Reference	Reference
Yes	181/231 (78.4)	2.51 (1.59–3.97)	1.36 (0.77–2.38)
Intention of belonging to *ikyoku*			
No	54/96 (56.3)	Reference	Reference
Yes	212/279 (76.0)	2.46 (1.51–4.01)	2.13 (1.25–3.63)
Interest in general practice/family medicine			
No	189/264 (71.6)	Reference	Reference
Yes	77/111 (69.4)	0.90 (0.55–1.46)	0.79 (0.46–1.35)
Recommending your program to high school students			
No	177/272 (65.1)	Reference	Reference
Yes	89/103 (86.4)	3.41 (1.84–6.32)	2.47 (1.24–4.92)
Satisfaction with education or activities specially designed for your program at medical school			
Not satisfied	170/247 (68.8)	Reference	Reference
Satisfied	96/128 (75.0)	1.36 (0.84–2.20)	0.96 (0.55–1.69)
Appreciation of the career development program			
No	124/191 (64.9)	Reference	Reference
Yes	142/184 (77.2)	1.83 (1.16–2.88)	1.24 (0.69–2.20)

*OR* odds ratio, *CI* confidence interval

## Discussion

The results of the present study indicate that interest in general practice/family medicine, life satisfaction, and intention of to belong to *ikyoku* were significantly associated with regional retention for medical students. Scholarship-bonded obligatory service was significantly associated with regional retention for CMPS students. Satisfaction with the career development program was also associated to some extent. For CMPS graduates, many of these factors lost their associations, and only satisfaction with training environment and intention of to belong to *ikyoku* remained associated. These results indicate the importance of improving the educational and training environment as well as the effective use of *ikyoku* for effective regional retention measures.

Students of CMPS were characterized by a higher proportion of women, a lower proportion of children of general practitioners, and a higher percentage of students whose prospects were medical specialties closely related to primary care than the other students. Regarding the lower proportion of children of general practitioners, this was due to the issue of tuition fees because most CMPS students were offered a scholarship. For the distribution of prospects of medical specialties, this finding may have arisen from the fact that CMPS students were more likely to choose specialties closely related to primary care. Furthermore, some of the regional quota programs were allocated to certain specialties considered to have shortages in the relevant prefectures, and this may have contributed to the difference. The overall decline in the percentage of respondents selecting prospect of medical specialty among graduates was due to the nature of multiple-response questions; in other words, the graduates were more determined in their career path than the medical students. It was noteworthy that the percentage of students choosing general practice was smaller among graduates than that of graduates. As the new board certification area for “General Practice” started recently, it is difficult to assess whether this difference in preference for general practice was due to a change in preferences as the school and postgraduate year progressed or whether it was among different generations.

In the present study, we identified several factors associated with regional retention after controlling for potential confounders. Identified factors such as satisfaction with their life and education and/or training environment and interest in general practice and family medicine were consistent with the findings in previous studies [2–4]. Systems unique to Japan, such as intention to belong to *ikyoku* and satisfaction with the newly introduced career development program, warrant further discussion.

Our results are noteworthy and provide lessons for not only Japan but also other countries. First, our results strengthen the findings of previous studies by identifying factors associated with physician retention in the community that may be generalizable to other countries under different systems compared with the countries where the studies were conducted. Second, the findings for the role of *ikyoku*, a system unique to Japan, clarified the importance of having a mechanism that achieves a sense of belonging and life-long education. The findings suggest that, regardless of different names for such mechanisms based on the context of field or history, a system for fostering a sense of belonging and life-long education is associated with regional retention. Therefore, even though *ikyoku* cannot be transplanted directly into another country, systems with functions similar to those of *ikyoku* could be applicable to other countries if they consider the local culture and history appropriately.

A previous study on physicians working in rural areas found strong correlations between intention to stay in the area and items such as interaction with local government, personal relationships, salary, and job satisfaction [24]. The provision of postgraduate education opportunities leads to continuation of work in remote areas as well as reduction of loneliness, which may be potential mechanisms for why belonging to *ikyoku* was associated with regional retention.

In the present study, for students with a scholarship-bonded rural service obligation, satisfaction with the newly introduced career development program was associated with regional retention. For graduates, this association was observed only in the crude analysis. This factor of career development program was only introduced recently when the guidelines were issued in 2018, and the program contents can vary. Even though the controlled OR for graduates did not show an association, the results are promising. Career coordinators, which were also introduced recently, may promote the function of a career development plan. Prefectures are required to appoint persons in charge of supporting physicians trained under the career development plan. These people are expected to assist eligible physicians by providing continuous support in rural and remote areas and career development such as board-certified specialist accreditation while they work in rural and remote areas.

Regarding undergraduate education activities in community medicine, one review study about the effects of community health education through undergraduate education indicated that longitudinal programs as an intervention were consistently associated with an increase in the proportion of students choosing primary care [25]. However, a retrospective cohort study in Japan did not support an association between the amount of undergraduate education for community-based medicine and subsequent increase in the number of general practice major residents [26]. Given the variety of contents of community health education at different universities, further examination of its effectiveness remains a challenge.

The present study has several limitations. First, the study had a cross-sectional design, and thus, the causal relationships remain unknown. Second, the survey asked respondents for their intention to settle in the region, and this intention does not guarantee their retention in the future. Third, the response rate of the study was not very high; thus, unexpected bias between respondents and non-respondents may exist.

Even with these limitations, the study was a nationwide large-scale survey that covered both students and physicians at the time when regional quota graduates are working in the community. The study provides useful information for assessing measures to address the maldistribution of physicians as well as identifying factors associated with physicians’ intention to stay in the community.

## Conclusions

In addition to known factors such as interest in general practice/family medicine, intention to belong to *ikyoku*, a Japanese traditional personnel system, was found to have a substantial impact on regional retention. The present results suggest that the career support system represented by *ikyoku* as well as a career development program are of potential importance for increasing regional retention through the mechanisms of a sense of belonging and a life-long education system. These findings provide useful information for developing further policy interventions that interweave traditional and new systems to maximize their effectiveness.

## Data Availability

The datasets generated and/or analyzed during the current study are available from the corresponding author on reasonable request.

## Abbreviations

JMU: Jichi Medical University
CMPS: Community medicine-oriented programs and schools
OR: Odds ratio
CI: Confidence interval
